# Metformin‐Mediated Glycaemic Regulation as a Potential Strategy for Breast Cancer Prevention

**DOI:** 10.1002/cam4.71573

**Published:** 2026-01-28

**Authors:** Ambulugala Gamage Rajika Greshamali Jinadasa, N. D. Amal Wageesha, Sameera R. Samarakoon, Sagarika Ekanayake, H. M. Kasuni Akalanka

**Affiliations:** ^1^ Department of Basic Sciences, Faculty of Allied Health Sciences University of Sri Jayewardenepura Nugegoda Sri Lanka; ^2^ Department of Biochemistry, Faculty of Medicine Sabaragamuwa University of Sri Lanka Ratnapura Sri Lanka; ^3^ Institute of Biochemistry, Molecular Biology and Biotechnology University of Colombo Colombo Sri Lanka; ^4^ Department of Biochemistry, Faculty of Medical Science University of Sri Jayewardenepura Nugegoda Sri Lanka; ^5^ Rural Health Research Institute Charles Sturt University Orange New South Wales Australia

**Keywords:** biochemical pathways, breast cancer, in vitro, metformin

## Abstract

**Introduction:**

Metformin, the common anti‐hyperglycemic agent, is emerging with pharmacological significance as an effective anti‐cancer modulator. Its efficacy as an anti‐cancer modulator is reported in pre‐clinical and clinical studies. Therefore, an attempt was made to identify the possible in vitro anti‐cancer molecular mechanisms studied on breast cancer (BC) cell lines.

**Methods:**

An advanced literature search was conducted in the PubMed database using search terms “Metformin, Cell culture, Breast neoplasms.” Different anti‐cancer molecular mechanisms induced by metformin (MET) identified in cell culture studies are presented in this paper.

**Results:**

It was identified that MET induces molecular pathways that exert anti‐cancer effects when treated on BC cells. Inhibition of oxidative phosphorylation, adenosine monophosphate‐activated protein kinase mediated anticancer effects, anti‐proliferation and inhibition of cell migration, alteration of tumor micro‐environment, synergetic effects with conventional chemotherapies and other potential molecules, induced apoptosis and ferroptosis were mainly identified as MET‐induced pathways that affect BC cells.

**Conclusion:**

Metformin induces diverse anti‐cancer biochemical pathways through which it exhibits a potential to be used as an anti‐cancer therapeutic in BC.

## Graphical abstract

Metformin (MET), a common antihyperglycemic drug, shows promising anti‐cancer effects in breast cancer (BC). It activates adenosine monophosphate‐activated protein kinase (AMPK), regulating cellular energy and inducing cell cycle arrest, anti‐proliferation, and microenvironment alterations. AMPK‐independent mechanisms include metastasis inhibition, drug synergism, ferroptosis, and apoptosis, highlighting MET's potential for novel BC therapies.

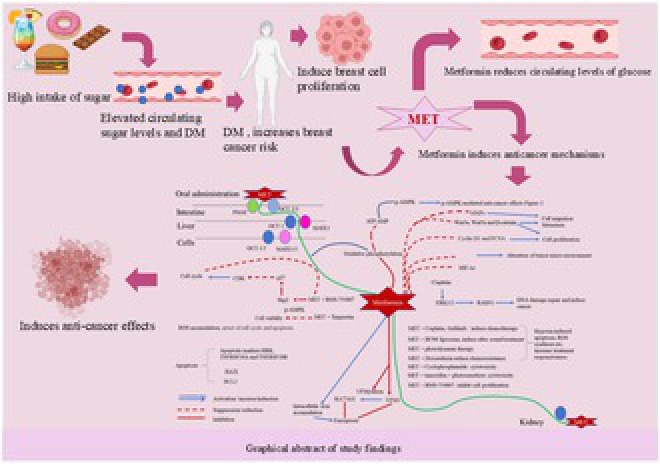

## 1 Introduction

An emerging trend is observed in repurposing metformin (MET), the most common anti‐hyperglycemic agent as a therapeutic option for cancer. MET is a synthetic biguanide. Biguanides were initially extracted from legume family plants for their glycaemic control properties [1]. However, these natural extractions were more prone to induce lactic acidosis, which led to the development of the synthetic analogue, metformin, with fewer side effects [2, 3].

The active pharmaceutical ingredient of oral MET is its hydrochloric form, metformin hydrochloride. Primarily, as a glycemic control agent, MET acts through adenosine monophosphate‐activated protein kinase (AMPK) mediated inhibition of hepatic gluconeogenesis and induction of peripheral cellular uptake of glucose in skeletal muscles [2] and adipose tissues. It is identified that MET is currently prescribed as the first‐line anti‐diabetic agent in control of Type 2 diabetes mellitus (DM) as it is the most effective molecule for glycemic control [4]. In addition to anti‐hyperglycemic properties, MET has demonstrated its effectiveness against different types of cancers including breast cancer (BC), highlighting its potential for a broader therapeutic application [2, 3, 4, 5, 6, 7].

Glucose is a primary source of energy for metabolically active cells [8]. Cells generate energy through oxygen‐dependent oxidative phosphorylation and/or glycolysis. Under physiological conditions, energy is predominantly generated through oxygen‐dependent oxidative phosphorylation, whereas limited oxygen availability shifts metabolism towards glycolysis and subsequent anaerobic respiration. In contrast, cancer cells preferentially utilize glycolysis to meet their adenosine triphosphate (ATP) demands even in the presence of adequate oxygen, a phenomenon known as the Warburg effect, which represents a hallmark of cancer metabolism. During glycolysis, multiple metabolic intermediates are produced that contribute to the biosynthesis of macromolecules essential for cellular proliferation. Given their rapid growth rates, cancer cells rely heavily on this glycolytic reprogramming to sustain continuous biomass synthesis and energy production. The capacity of cancer cells to adapt to oxygen‐deprived microenvironments further enhances their ability to survive under tissue hypoxia [9, 10, 11]. Such metabolic flexibility promotes tumor progression, therapeutic resistance, and survival under adverse conditions. Following hypoxia, cells induce the accumulation of hypoxia regulatory factor 1 (HIF‐1), a transcription factor that induces cell survival through metabolic changes. It is a hallmark of the Warburg effect and induces glycolytic pathways via upregulation of GLUT1/3, hexokinase (HK), pyruvate kinase M2 (PKM2), and lactate dehydrogenase (LDH). This HIF‐1 also inhibits mitochondrial respiration via pyruvate dehydrogenase kinase 1 (PDK1) [11]. These metabolic alterations collectively enable cancer cells to maintain energy homeostasis, resist apoptosis, and proliferate within glucose‐limited and hypoxic tumor microenvironments.

Among various carcinomas, BC exhibits the highest incidence and prevalence globally among women, although it generally carries a comparatively favorable prognosis. Epidemiological studies have demonstrated that DM increases the relative risk of BC by approximately 10%–20% [12, 13]. Several in vitro, preclinical, and clinical studies reported the efficacy of using MET individually as an anticancer agent or in combination with chemotherapeutic drugs or radiation in the treatment of different forms of BC. The BC patients with DM who receive MET during their neoadjuvant chemotherapy have shown a higher pathologically complete response rate than DM patients not receiving metformin. The metabolic effect of MET begins with the inhibition of mitochondrial complex I, which reduces cellular ATP levels and consequently increases the AMP/ATP ratio. This energy stress activates AMP‐activated protein kinase (AMPK), a central metabolic sensor that suppresses cell growth and proliferation [14]. In cancer cells, MET attenuates the Warburg effect by decreasing glucose uptake. It is achieved through decreasing glycolytic enzyme expression and reducing lactate production [15]. In BC, MET disrupts the glycolytic and biosynthesis pathways which are driven by HIF‐1 activation [16]. Preclinical and clinical trials have identified that MET improves the response rates of chemotherapy and conventional endocrine therapies in metastatic BC [17]. These combined molecular, metabolic, and translational effects of MET mark it as a potential anticancer therapeutic and enhancer of therapeutic outcome for BC [8, 13, 18, 19]. Given the ongoing debate regarding the pharmacological significance of MET in cancer, this review summarizes the reported molecular mechanisms underlying its potential anti‐cancer effects on BC cells.

### 1.1 Cellular Uptake of Metformin

Metformin is a hydrophilic compound and remains ionized at physiological pH. Following oral administration, its cellular uptake occurs via transmembrane transporters. Plasma membrane monoamine transporter (PMAT) and organic cation transporters 2 and 3 (OCT2/3) facilitate the uptake of MET in enterocytes. Hepatic uptake of MET is mediated by OCT1 and multidrug and toxin extrusion protein 1 (MATE1), whereas renal uptake occurs through renal epithelial OCT2 [20].

Similar cellular transporters are shown to be involved in the uptake of MET in in vitro BC cell line. MCF7 cells expressing higher levels of organic cation transporters (OCTs) exhibit nearly threefold greater cellular uptake of MET compared with MCF7 cells expressing lower OCT levels [21]. Markowicz‐piasecka et al. reported a significantly higher abundance of OCT3 transporters in MCF7 cells than in MDA‐MB‐231 cells, facilitating increased MET uptake, which correlates with enhanced antiproliferative activity in MCF7 cells [20]. MATE1 transporters are expressed in both MCF7 and MDA‐MB‐231 cell lines, whereas MATE2 expression is absent. Additionally, PMAT expression is approximately threefold higher in MCF7 cells compared with MDA‐MB‐231 cells [20].

The availability and expression profile of these transporters play a crucial role in facilitating MET uptake and directly influence its intracellular pharmacological effects.

Nanoencapsulation has been explored as a strategy to enhance the efficiency of cellular uptake. In one study, MET was embedded in nano particles and radiolabeled with technetium‐99m tricarbonyl core, which resulted in greater cellular uptake compared with free MET in both MCF7 and MDA‐MB‐231 BC cell lines. Among these, MCF7 cells exhibited higher uptake than MDA‐MB‐231 [22]. Similarly, nanoencapsulation of MET using an O‐carboxymethyl chitosan (O‐CMC) polymeric formulation demonstrated comparable cellular uptake and anticancer efficacy in MCF7 cells, while maintaining biocompatibility in MCF‐10A non‐carcinogenic breast epithelial cells [23]. Collectively, although MET enters cells primarily through transmembrane transporters, its therapeutic potential as an anticancer agent can be further improved through nanocarrier‐based delivery systems. Such approaches enhance bioavailability, targeted delivery, and intracellular retention, ultimately leading to improved therapeutic outcomes.

## 2 Mechanisms of Action

### 2.1 Adenosine Monophosphate‐Activated Protein Kinase Mediated Anticancer Effects

Adenosine monophosphate‐activated protein kinase is identified as the master regulator of cellular energy levels [24].

**FIGURE 1:**
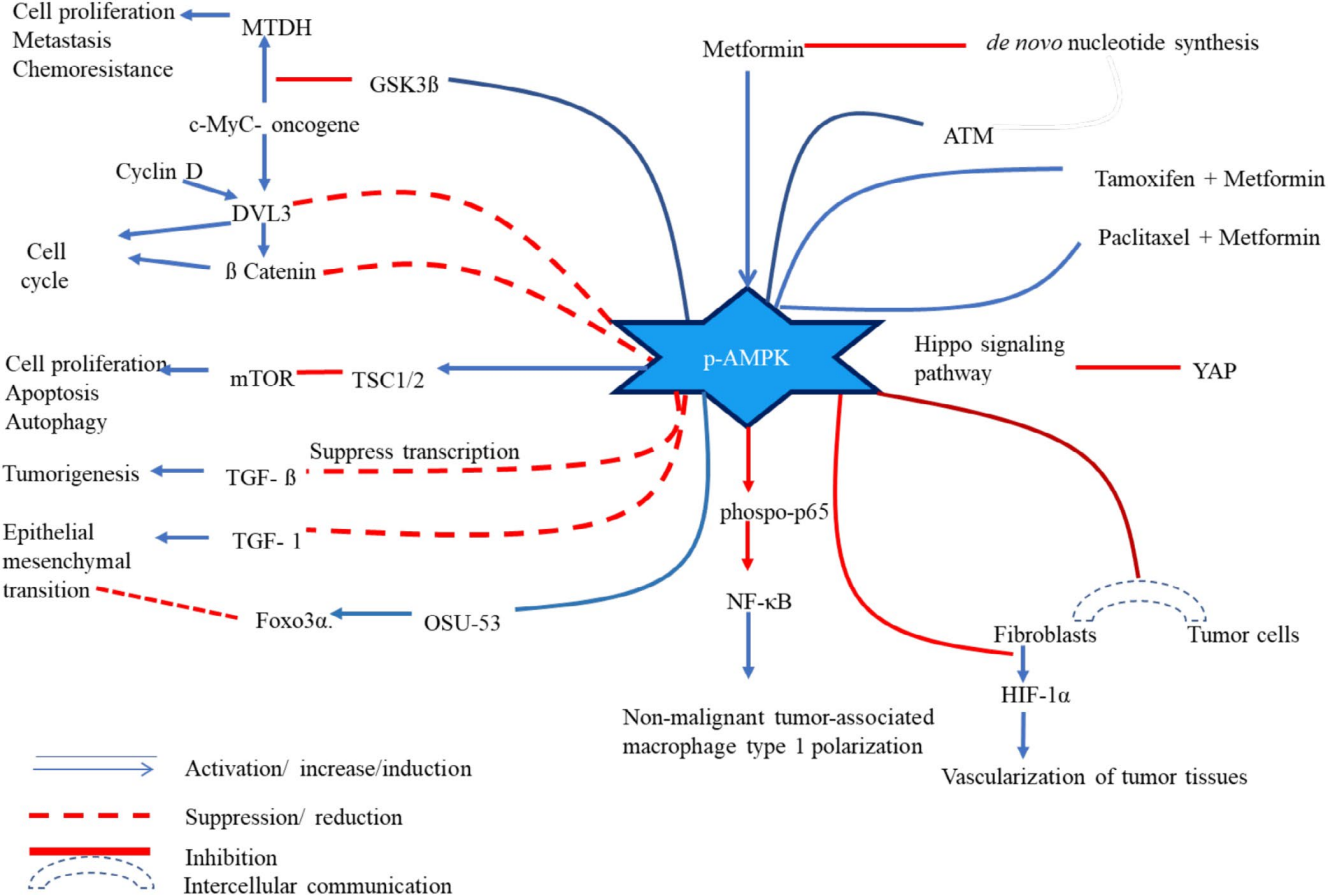
Anticancer effects of activated adenosine monophosphate kinase. AMPK, adenosine monophosphate kinase; ATM‐ataxia, telangiectasia‐mutated protein kinase; c‐Myc, myelocytomatosis; DVL, disheveled segment polarity protein 3; EMT, epithelial–mesenchymal transformation; HIF‐1α, hypoxia‐inducible factor‐1α; MET, metformin; MTDH, metadherin; mTOR, mammalian target of rapamycin; NF‐κB, nuclear factor‐κB; TGF‐ß, transforming growth factor‐ß; TSC, tuberous sclerosis complex; YAP, yes‐associated proteins.

This molecule plays a pivotal role in maintaining the anticancer mechanisms of MET. MET's primary cellular target is mitochondria. It is a known inhibitor of complex I in the electron transport chain of the inner mitochondrial membrane [25]. This knockdown of the cellular energy metabolism yields a decreased cellular ATP and increased AMP, thus altering the AMP:ATP ratio. Increased AMP:ATP ratio activates AMPK. Activated AMPK directly and indirectly acts on cellular pathways and induces different anticancer mechanisms as illustrated in Figure 1.

These include anti‐proliferative effects, induced apoptosis, cell cycle arrest, downregulating tumorigenesis, altering epithelial–mesenchymal transformation (EMT), and rearranging the tumor microenvironment. Anti‐proliferative mechanisms mediated by AMPK are evident by the following.

Activated AMPK downregulates Metadherin (MTDH), an oncogenic protein that induces cell proliferation and metastasis [26]. This action is mediated via glycogen synthase kinase 3β (GSK3β). The MTDH is activated by myelocytomatosis (c‐Myc), where c‐Myc is a master oncogene that codes for cell proliferation and apoptosis regulatory proteins. Cells treated with MET *have been observed to accumulate* 5‐formimino‐tetrahydrofolate, a one‐carbon carrier required for de novo purine and pyrimidine synthesis, *indicating* disruption of nucleotide synthesis through the anti‐folate activity of MET. This pathway is *secondarily influenced* by AMPK activation, as AMPK *stimulates* the activity of ataxia‐telangiectasia mutated (ATM) protein kinase. ATM, a key regulator of the DNA damage response, *retards* progression towards the invasive phase of carcinoma and also acts as an upstream kinase for AMPK. Therefore, MET *exerts antiproliferative effects* via AMPK‐mediated anti‐folate and tumor‐suppressor pathways [27]. In another mechanism, disheveled segment polarity protein 3 (DVL3) upregulates the β‐catenin in the Wnt/β‐catenin pathways and c‐Mya induce in vitro cell proliferation of BC cells. The activated AMPK is observed to downregulate DVL3 and β‐catenin, inhibiting the cell cycle and ultimately resulting in cell cycle arrest [28]. Cell proliferation is facilitated by profound protein synthesis, particularly in cancer cells.

The master controller responsible for protein synthesis in cells is known as mechanistic target of rapamycin or the mammalian target of rapamycin (mTOR). Activated AMPK is identified as a negative regulator of mTOR [29]. This negative controlling effect is mediated via the tuberous sclerosis complex 1/2 (TSC1/2) pathway [30]. The TSC1 is a component of the TSC1/2 complex. TSC1 converts Rheb, a GTPase in the mTOR pathway, to its inactive form, GDPase‐bound form. The GTPase‐bound form activates mTORC1 and promotes cell growth and proliferation. Whereas, inactive GDPase inhibits the mTOR pathway. In addition, combined treatment of MET with urosilic acid and MET with everolimus has demonstrated AMPK‐mediated inhibition of mTOR [30, 31].

Cancer cells are known to alter their cellular structures and undergo malignant transformation. These transformations take place in various cellular components, including cell receptors, chromatin content, and proteins.

A specific type of structural change, known as EMT, enables transformed cells to withstand metastasis and enhance their survival, ultimately promoting tumor progression in later stages.

TGF‐β phosphorylates SMAD 2/3 and facilitates its transcription to support EMT. SMAD proteins are intracellular proteins that mediate signals from TGF‐β. However, liver kinase B‐1 (LKB‐1) and AMPK have demonstrated suppression of TGF‐β mediated phosphorylation of SMAD 2/3 and prevented EMT in MDA‐MB‐231 cells [32, 33]. Additionally, activated AMPK mediates akt/dependent pathways where Foxo3α and OSU‐53 suppress EMT [24]. However, uncontrolled cell proliferation and cellular transformation rearrange the cellular environment for tumorigenesis and metastasis of tumors. Accordingly, activated AMPK demonstrates antiproliferation, cell cycle arrest, and cellular transformation.

In addition to affecting cellular processes and altering cellular transformation, activated AMPK modifies the tumor microenvironment, which is another crucial hallmark of invasive cancer. Tumor‐associated macrophages consist of two distinct subpopulations that influence tumor progression. These macrophage subtypes are M1 and M2. The M1 type acts as an anti‐tumor scavenger, whereas M2 macrophages promote tumor growth and malignancy.

MDA‐MB‐231 bc cells were cultured in the presence or absence of MET. Their conditioned media (CM) following 6‐h treatment were subsequently used to culture THP‐1 human monocytic cells for 24 h. Gene expression profiling of THP‐1 cells revealed M1 macrophage polarization in the presence of MET‐treated CM, while M2 polarization was observed in untreated CM‐grown cells. MET‐treated BC cells secreted pro‐inflammatory cytokines, including IL‐12 and TNF‐α, that promote the M1 phenotype, while the levels of IL‐4, IL‐10, and IL‐13, M2 polarization‐associated cytokines, were dramatically reduced. The macrophage phenotypic switch was regulated by AMPK‐dependent inhibition of NF‐κB, an overexpressed transcription factor complex in the majority of cancers that regulates cytokine secretion, inflammation, cell growth, apoptosis resistance, and oxidative stress [34]. These findings indicate that MET induces anti‐tumor immunity in the tumor microenvironment by modulating macrophage polarization and its anticancer activity in the microenvironment of BC is thus highlighted.

Vascularizing of the tumor micro‐environment is essential for preventing ischemic cell death and supporting the survival of cancer cells within tissues. Cancer associated fibroblasts express hypoxia‐inducible factor‐1α (HIF‐1α) on their cell membrane, which induces vascularization of the tissue. MET‐mediated AMPK inhibits the expression of HIF‐1α on fibroblasts [35]. Therefore, it is revealed that the majority of the anti‐cancer effects of MET are mediated via AMPK activation.

Another anticancer biochemical pathway is regulated through the yes‐associated protein (YAP) involved pathway. This YAP is a transcriptional coactivator and a regulator of the Hippo pathway (HPP). In its inactive state, the YAP protein is phosphorylated and retained in the cytoplasm, whereas dephosphorylation activates YAP, leading to its nuclear accumulation. Within the nucleus, YAP activates genes involved in cell growth and proliferation, particularly in cancer cells. Nuclear accumulation of YAP has been observed in drug‐resistant BC, where it contributes to tumor progression and therapeutic resistance. When activated, the molecule is acting as a mechanotransducer, which induces intracellular biochemical signals via gene expression in response to extracellular mechanical signals such as tension and compression. In a malignant environment, when tissue growth is compromised, YAP is activated as an oncogene to induce cell growth, proliferation, and survival. In the HPP, large tumor suppressor 1 and 2 (LTS1/2) and angiomotin‐like 1 (AMOTL1) are upstream components and phosphorylate YAP. Phosphorylation ensures the inactive cytoplasmic retention of YAP and prevents nuclear transcription. AM PK has been identified as directly phosphorylating AMOTL1 at Ser793. Thus, AMPK indirectly inhibits YAP activation [36]. Another noteworthy cell proliferation inhibition is associated with YAP regulation. Activated AMPK directly phosphorylates the Ser94 residue of the YAP protein, thereby suppressing its transcriptional activity and inhibiting YAP‐mediated cell growth. However, AMPK‐independent YAP inactivation is also observed and is discussed under other signaling pathways [37]. The combination of MET with paclitaxel and MET with tamoxifen has induced combined anti‐cancer efficacy via phosphorylated AMPK mediated pathways [38, 39]. Figure 1 summarizes the AMPK mediated anti‐cancer mechanisms described above.

### 2.2 Anti‐Proliferation and Inhibition of Cell Migration

Uncontrolled cell proliferation and abnormal cell growth are hallmarks of malignant transformations. Designing therapeutics targeting these cell proliferation pathways is therefore common. MET has been identified as an effective molecule for inhibition of malignant cell proliferation and metastasis. The Wnt is a cell signaling pathway. It is a crucial pathway in regulating cell proliferation, differentiation, migration, and survival. In normal physiology, the Wnt pathway is involved in stem cell maintenance in adult tissues and embryonic development. Wnt proteins are cell surface signaling molecules which activate intracellular signaling cascades. The activation of the Wnt pathway is aberrantly seen in cancer, and it has several pathways of activation. The commonest is the binding of Wnt with β‐catenin. β‐catenin is a protein which is stabilized when bound to Wnt. The stabilized Wnt‐β‐catenin complex enters the nucleus and promotes gene expression. Uncontrolled Wnt‐β‐catenin signaling initiates atypical cell proliferation. When MCF7 cells were treated with MET, the expression of Wnt3a, Wnt5a, and β‐catenin have been significantly downregulated [40]. Unrestrained cell cycle progression is a vital checkpoint in carcinogenesis. Assessing the expression of cell cycle biomarkers is another reliable evidence in finding anti‐cancer effects of drugs. Cyclin D1 and proliferating cell nuclear antigen (PCNA) are two such markers of the cell cycle. Cyclin D1 status regulates cell cycle transition from G1 to S phase. It also activated cyclin dependent kinases (CDKs) such as CDK 4/6. Similarly, PCNA assists DNA polymerase enzyme during DNA replication. In cancer, when the cell cycle is uncontrolled and rapid proliferation occurs, elevated levels of cyclin D1 and PCNA are observed. When drug‐sensitive MCF7, Tamoxifen (TAM)‐resistant cells LCC2 and paclitaxel (TAX) resistant MCF7/TAX were treated with MET, reduced levels of cyclin D1 and PCNA were observed [37].

Lymph node and brain metastasis are common features of invasive BC. The effect of MET in preventing tissue metastasis represents an important aspect of its anticancer activity. Treatment of MCF7 BC cells with MET has been shown to downregulate key biomarkers of metastasis. Matrix metalloproteases (MMPs) are a group of enzymes such as collagenases, gelatinases, and matrilysins. These MMPs digest the extracellular matrix and promote metastasis in cancer [41]. Experimental evidence has reported decreased expression of MMPs when BC MCF7 cells were treated with MET. This indicated the MET driven prevention against metastasis. Metformin inhibited MCF‐7 cell growth dose‐dependently, with significant effects at 10 and 50 mM. High‐dose metformin significantly inhibited MMP‐2 and MMP‐9 expression and interfered with NF‐κB (p65) localization, turning towards elevated cytoplasmic levels and diminished nuclear translocation, thereby suppressing pro‐proliferative and pro‐metastatic signaling. These effects are predominantly bestowed through AMPK activation and mTOR inhibition, leading to cell cycle arrest. Metformin overall appears to be a potential anti‐proliferative and anti‐metastatic drug in BC in vitro [42]. The scratch assay or the cell migration assay is an in vitro protocol to assess potential metastasis of cancer cells. When scratch assay was performed on MET treated triple negative MDA‐MB‐231 cells, impaired cell migration was observed. This also indicates potential anti‐metastatic features of MET on BC cells [19].

### 2.3 Alteration of Tumor Micro‐Environment

Survival of any cell is supported by its neighboring cells as well as the surrounding micro‐environment. In malignant transformation, changes occur within the cell itself as well as its surroundings.

Adipose derived stromal cells (ADSC) are defined as a group of multipotent, undifferentiated, self‐renewing progenitor cell populations that are morphologically and phenotypically similar to bone marrow mesenchymal cells. The abdomen and breast are sources of ADSC. It is stated that ADSCs are responsible for promoting growth, progression, and metastatic spread of residual or de novo BC after resection. Following mastectomy, ADSCs activate any residual un‐resected microscopic tumor foci and promote BC progression. Furthermore, according to Schweizer et al., ADSCs have the potential for assisting the malignant transformation of breast tissues. When the CM of ADSC was observed to stimulate the growth and survival of MDA‐MB‐231 cells [43]. Interestingly when the MET treated CM of ADSC was used to grow MDA‐MB‐231 cells, the growth was shifted towards inhibition, indicating the MET mediated attenuation of malignant effects of ADSC [19]. Therefore, it is evident that MET is demonstrating reverse effects of tumor environment mediated carcinogenesis.

Supply of adequate oxygen to the tissue environment is an absolute requirement for the survival of cells. Hypoxia is commonly observed in cancer and is evidence of mutagenesis in cancer cells. Cancer cells induce hypoxia intermittently and produce hypoxia inducible factors (HIF). This HIF promotes the transcription of a large variety of genes that encode for different survival mechanisms of cells. Among such, tumor vascularization, cell growth, remodeling of the tumor micro‐environment, metastasis and tissue invasion, alteration of cell mortality and stromal cell adaptations are crucial and aid in cancer progression. In addition, hypoxia is known to induce drug resistance in BC. It is crucial to define hypoxia in cancer since hypoxia is concurrently associated with non‐malignant cellular mechanisms such as apoptosis, inflammation and necrosis [44]. Hypoxia is frequently observed in invasive cancer phenotypes in the breast, pancreatic and prostate while leading to therapeutic resistance. Among BC phenotypes, TNBC is an invasive subtype which frequently gets affected by hypoxia.

About 50%–60% of solid tumors are experiencing a hypoxic microenvironment and reduce the efficacy of treatment strategies. In addition to chemotherapy, sono‐dynamic therapy (SDT), which is less invasive and has deeper penetration capabilities, is emerging over conventional treatments. However, tissue hypoxia has been observed to compromise the effectiveness of SDT as this treatment is oxygen‐sensitive. The utilization of tissue oxygen by inhibiting cellular oxygen uptake was observed to be more effective in upregulating the therapeutic effect of SDT. In contrast to the above anti‐hypoxic properties, the mechanistic properties of MET have been used to elevate the tissue oxygen level to improve the effectiveness of SDT. MET is known for its mitochondrial effect [43]. Inhibition of oxidative phosphorylation by MET has been successfully employed in maintaining oxygen levels of the tumor microenvironment. MET inhibits the activity of mitochondrial complex I, thereby controlling tissue oxygen consumption and designing a favorable environment for SDT effectiveness. A nanoencapsulation of a pH dependent drug delivery complex loaded with MET has been tested on MDA‐MB‐231 bc cells. The cells were incubated in an underoxygenated environment, treated with the above liposome, and exposed to ultrasound (US) treatment. The results demonstrated an increased cytotoxicity, accumulation of reactive oxygen species, pH dependent MET release, and increased bioavailability have improved the effectiveness of US treatment on the BC cells [45]. A similar study has identified that a nano‐carrier of MET and catalase enzyme has improved the tissue oxygen levels, thus improving the effectiveness of the photosensitive therapy. The study was conducted in cellular human BC (Bcap37) cells [46]. The combined use of MET with chemotherapy, SDT, and photosensitive therapy has improved the effectiveness of the combination in anti‐cancer treatment.

### 2.4 Synergetic Effects

Synergism is when two or more drugs combinedly exhibit greater effect than predicted by their individual potency [47]. MET is observed to exhibit synergetic effect with different drugs where some of the examples were discussed above under different mechanisms. In addition, the following synergisms of MET are also observed as treatment options for BC: the synergistic effects of MET are observed with known conventional anti‐cancer drugs, natural compounds and biomolecules with potential anti‐cancer effects.

Oxidative stress and inflammation related pathways were observed as chemo‐resistant mechanisms in MCF‐7 and MDA‐MB‐231 bc cells. Doxorubicin is known to cause chemo‐resistance due to oxidative stress. BC cells pretreated with 6 μM of MET were observed to be less chemoresistant to doxorubicin [48]. The synergistic action has been tested in a nano encapsulated doxorubicin and metformin particle [49, 50, 51]. Increased cytotoxicity, growth inhibition, glucose deprivation, synthesis of ROS by MET alone and synergistic effect of MET with doxorubicin by downregulating drug resistant genes in MCF‐7 cells had been observed [51]. In the co‐delivery of doxorubicin and MET, MET suppresses HIF1α and P‐glycoprotein (Pgp) to reduce tissue oxygen consumption thus support the cytotoxic effect of doxorubicin [49].

Cisplatin is used in the treatment of TNBC. Cisplatin binds to purines adenine and guanine of DNA to form crosslinks between the molecules and damage DNA. Cells terminate their life with apoptosis [52]. The use of cisplatin in combined therapy is observed in cancer treatment including TNBC [53, 54]. However, cisplatin has been observed to induce interleukin‐6 (IL‐6), and in turn IL‐6 induces HIF‐1, drug resistance, and cancer stem cell progression via NK‐kB and STAT3 mediated pathways [55, 56]. Increased hypoxic conditions stimulate drug resistance. When TNBC cells were treated with cisplatin hypoxia, it induced transcription of HIFs. Also, meanwhile, it induced drug resistance. When cisplatin was combined with MET and gefitinib, the activity of HIF was reduced and drove the cells to apoptosis [56].

Chemoresistance frequently emerges in invasive BC, particularly with prolonged chemotherapy exposure. Among standard chemotherapeutic agents, cisplatin has been reported to exhibit synergistic effects when combined with metformin (MET). Studies assessing cell viability, proliferation, migration, and invasion in MET‐sensitized MDA‐MB‐231 cells followed by cisplatin treatment have demonstrated enhanced anticancer efficacy. The molecular mechanism behind this synergistic activity is mediated via RAD51 expression. RAD51 is a protein that repairs DNA breaks in double‐stranded DNA. In cancer cells, the expression of RAD51 improves their survival and cancer progression. Cisplatin is observed to upregulate the level of RAD51 in TNBC (MDA‐MB‐231 and Hs 578T) and the normal breast cell line MCF10A. In contrast, western blot analysis has revealed that the treatment of cells with MET has downregulated RAD51 in both TNBC and normal breast cell lines. The combined treatment of cells with cisplatin and MET has interestingly downregulated the expression of RAD51 by decreasing the stability of the protein via proteasomal degradation. In addition to these mechanisms, RAD51 expression is known to be mediated via extracellular signal‐regulated kinases 1/2 (ERK1/2) which is induced by cisplatin. However, MET inactivates the phosphorylation of ERK1/2 and exhibits anti‐cancer properties. Further MET has revealed Cisplatin mediated DNA damage through H2AX signaling. H2AX is a histone protein. Its phosphorylated form γ‐H2AX is a marker of double‐stranded DNA damage response. MET when combinedly treated with cisplatin has upregulated the level of H2AX than treatment with cisplatin alone. This indicates that MET when combined with cisplatin synergistically induces DNA damage in TNBC [57]. Collectively, these findings demonstrate a favorable synergism between MET and cisplatin, highlighting their potential as a therapeutic combination for overcoming chemoresistance in TNBC.

MET has also been identified as an effective adjuvant therapy. Cyclophosphamide (CP) is an anti‐neoplastic agent for cancers including BC [58]. The drug CP is known for its serious bladder dysfunction and hemorrhagic cystitis [59]. The TNBC, MDA‐MB‐231 cell lines were treated with 0.1, 1, and 10 mM of MET. Among these concentrations, 1 mM has augmented the cytotoxicity of CP. The MTT cell viability assay has proven this augmented cytotoxicity effect of CP and MET [60]. This suggests anti‐neoplastic synergism of MET with CP.

Cancer treatment methods are not confined to chemotherapy. Treatment sometimes includes a combination of treatment strategies. Photodynamic therapy is one such treatment option which is a non‐invasive treatment option. The therapy has been evaluated for synergetic effect with MET. A synthetic coelenterazine base photosensitizer has been designed to self‐activate intracellularly via a chemiluminescent process which is triggered by a cancer marker. Herein, co‐treatment with 1.27 μM MET, 2.22 μM TAM, a well‐established anticancer drug, and the photosensitizer demonstrated enhanced anticancer efficacy, as evidenced by reduced cell viability and increased cytotoxicity [61].

Insulin‐like growth factor 1 receptor (IGF1R) and insulin receptors (IR) induce tumorigenesis. These receptors activate I3‐Kinase, Akt, and Ras, which are known potent oncoproteins that are deregulated in many cancers. The inhibition of IGF1R revealed anticancer effects with reduced cell viability. BMS‐754807 is a pyrrolotriazine that is identified as an inhibitor of IGF‐1R and IR. A study on synergistic effect of BMS‐754807 (15 μM) and MET (5 μM) for the combined anticancer effect on TNBC the cell proliferation was measured using a methane thiosulfonate‐based assay. This assay revealed more than 50% inhibition of cell proliferation. Among 13 different TNBC cell lines 11 demonstrated sensitivity towards the above combination. Cell lines such as BT‐20, HCC1806, MDA‐MB‐436, HCC70, and MDA‐MB‐468 have shown significant sensitivity to the combined anti‐cancer effect. As per reverse‐phase protein array when treated alone, MET alters the action of 35 proteins, while BMS‐754807 alters 118 proteins. In combined treatment when MET was added to BMS‐754807 and vice versa 51 more alterations and 121 more protein alterations were seen respectively indicating synergistic activity in protein alteration. The altered proteins play significant roles in different anti‐cancer pathways. The enhanced p27KIP1 and its phosphorylated form were significant inhibitors of the CDK complex.

It is phosphorylated at Thr187; accumulation of p27 causes cell cycle arrest. The combination effect results in accumulation of p27 leading to cell cycle arrest in G1 phase, which stops cell cycle progression. This accumulation of p27 protein is mediated via the Skp2 involved pathway [62].

Synergistic effect of MET, acetylsalicylic acid (ASA), and oseltamivir phosphate (OP) was studied in MDA‐MB‐231 cells. MET and ASA in the concentration range of 0.5–16 mM and OP in the range of 25–800 μg/mL were tested on MDA‐MB‐231. It was revealed that these compounds are effective in inducing anti‐cancer effects on TNBC cells in a dose‐dependent manner. Furthermore, combination of ASA (8 mM), MET (4 mM), and OP (300 μg/mL) caused a significant (*p* < 0.0001) decrease in viability of MDA‐MB‐231 cells together with tamoxifen (TAM) in a dose‐dependent approach. The combined treatment increased necrosis of cells. Expression of high CD44 and low CD24 (CD44^+^/CD24^−/low^) is a marker of chemo‐resistance and enhances invasion and metastasis [63]. When treated alone, MET, OP, TAM, and ASA have shown increased (CD44^+^/CD24^−/low^) ratio. This CD marker acquisition is similar to a transformation into a cancer stem cell. Cancer stem cells promote invasion and metastasis of cancer. When TAM is treated alone, the CD marker ratio is 55; in untreated cells, it is 13. Then TAM is combined with MET, ASA and OP the ratio is 2. This indicates the potential reduction of the stemness of TNBC in this combination of drugs [64]. A similar study has identified the synergic effect of MET in combination with TAM, Trastuzumab, and anti‐ROR1 on BC cell lines by downregulating proliferation, colony formation, migration, invasion, and tumor growth. Combination therapy was effective at non‐toxic levels and inhibited tumor growth and metastasis in ex ovo CAM assays. Mechanistically, MET activates AMPK and suppresses PI3K/AKT/mTOR, RAS/MAPK, and Wnt pathways, whereas targeted therapies suppress ER, HER2, or ROR1 receptors. Cumulatively, these effects synergistically inhibit cell survival, angiogenesis, EMT, and inflammation, demonstrating the therapeutic potential of metformin to augment targeted BC treatments [65]. As stated earlier, beyond the conventional chemotherapies and cancer treatment methods, MET acts synergistically to induce anticancer effects with natural compounds and biomolecules. According to Mdkhana et al., tangeretin and MET synergistically act as anticancer agents [66]. Tangeretin is a naturally occurring citrus methoxyflavone with potential anticancer effects. Tangeretin was observed to improve the anticancer effect of MET. The cell viability studies evaluated using MTT assay using MCF‐7 and MDA‐MB‐231 cell lines have revealed synergism in an AMPK‐mediated pathway and generation and accumulation of ROS leading to cell death. This synergism is more effective in the resistant BC variants. In addition, increased arrest of cell cycle and apoptosis is upregulated in this synergism.

Bio‐molecule crocin, which is used for prevention of metastasis, when evaluated, revealed cytotoxic, anti‐invasive, and anti‐adhesive effects of MET and crocin on 4T1 cell lines in in vitro studies [67]. The co‐effect of Sulforaphane and nano‐MET has been studied and revealed that the higher HER 2 level in the BC cells shows increased cellular uptake. These compounds together reduce the cancer stem cell signaling and CD44 mediated carcinogenic effects in HER2 positive cells [68].

In addition, MET and rapamycin induce cell cycle arrest in BC cells. MET was observed to induce G2/M arrest and oxidative stress, while rapamycin induced G0/G1 arrest in ER+ cells. Proteomic and modeling analysis indicated alterations in mitochondrial function, ROS enzymes, and metabolic pathways, and single nucleotide polymorphisms had an impact on drug sensitivity. This combined strategy illustrates how the integration of experimental and modeling strategies can disclose drug mechanisms and metabolic weaknesses in BC [69].

Therefore, chemotherapy and antimetabolic drugs inhibited the growth of TNBC cells in cell line‐ and genetic background‐dependent manners. MET and 2‐D‐deoxy‐glucose, targeting glycolysis, synergized with chemotherapy and with each other, while antiglutamine drugs had cell‐dependent effects. The combinations reduced ATP, induced apoptosis, and were comparatively non‐toxic, indicating the potential for metabolic targeting in the treatment of TNBC [70].

### 2.5 Induced Apoptosis and Ferroptosis

Programmed cell death or apoptosis is a strong anti‐cancer mechanism induced by compounds tested on cancer cells. Evidence of MET mediated apoptosis is also available. Treating of BC cells with MET has induced apoptosis markers namely HRK, TNFRSF10A and TNFRSF10B indicating apoptosis induction by MET [62]. Ferroptosis is a newly defined form of cell death where apoptosis is induced by iron ions [71]. During oxidative phosphorylation, iron is involved in the synthesis of reactive oxygen species (ROS) in mitochondria. Iron ions dependent intracellular accumulation of ROS causes cellular damage and leads to programmed cell death; thus, this newly suggested mechanism of cell death is termed as ferroptosis [67]. In the context of cellular metabolism, MET is known as an iron regulator [72]. Thus, MET, when treated on MCF7 and T47D cells, increased the level of intracellular iron ions [73]. The mechanism of increasing iron level is yet unrevealed. Concurrently elevated iron ions stimulate the cellular environment to accumulate ROS, suggesting the potential cell death. Following the treatment of cells with MET (5 mM), reduced cellular viability together with elevated iron levels manifest the potential activation of ferroptosis in BC cells [73].

The treatment of drug sensitive and drug resistant BC cells with MET revealed induction of apoptosis. This was evident with cleavage of caspases 3, increased expression of BAX and reduced BCL2 expression [37]. Therefore, it is evident that MET is acting as an apoptosis and ferroptosis inducer.

The molecular mechanism of ferroptosis is triggered by lipid peroxidation, which is regulated by SLC7A11 protein. SLC7A11 is a membrane protein in the glutamate cystine antiport and regulates the synergetic lipid peroxidation by reduced glutathione (GSH) and glutathione peroxidase 4 (GPX4), followed by inhibition of ferroptosis and aid in cell survival. The treatment of MCF7 and T47D cells with MET has inhibited the expression of SLC7A11 in a posttranscriptional manner, triggering the cellular environmental changes towards induced ferroptosis [73].

Furthermore, the activity of SLC7A11 is regulated by UFM1 which is a ubiquitin‐like modifier (UBL) in the process of UFMylation. UFMylaion is a protein modification process. Where, a ubiquitin‐fold modifier 1 (UFM1) is attached to the target protein. These UBLs attach onto eukaryotic proteins, post‐translationally, and regulates the cellular mechanisms [74]. The UFM1 is involved in the incidence and progression of BC. MET has indirectly reduced the level of UFM1 protein in the cellular environment and downturn the UFMylation process. The level of UFM1 promotes BC by two mechanisms; directly by restoring the inhibition of GSH and stimulating ferroptosis and indirectly by inhibiting the UFMylation of SLC7A11. Therefore, MET reduces the activity of SLC7A11 by inhibiting UFMylation and influencing the level of GSH [73].

### 2.6 Other Mechanisms

**FIGURE 2:**
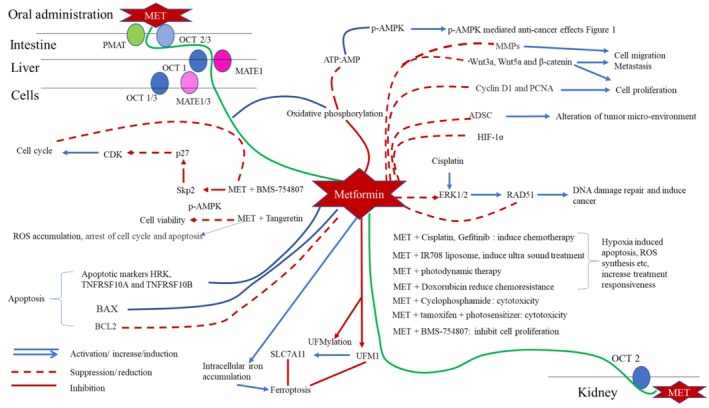
Anticancer effects of Metformin. ADSC, Adipose derived stromal cells; AMP, adenosine monophosphate; AMPK, adenosine monophosphate kinase; ATM‐ataxia, telangiectasia‐mutated protein kinase; ATP, adenosine triphosphate; CDK, cyclin dependent kinase; c‐Myc, myelocytomatosis; EKR, extracellular signal‐regulated kinases; HIF‐1α, hypoxia‐inducible factor‐1α; MATE, multidrug and toxin extrusion; MET, metformin; MMPs, matrix metalloproteases; MTDH, metadherin; OCT, organic cation transporters; PCNA, proliferating cell nuclear antigen; PMAT, plasma membrane monoamine transporters; ROS, reactive oxygen species; TSC, tuberous sclerosis complex; UFM1, ubiquitin‐like modifier 1.

Drug‐resistant BC are known for their poor prognosis and low response rates. Treatment options for drug‐resistant BC include endocrine therapies such as tamoxifen (TAM), aromatase inhibitors (anastrozole), chemotherapeutic drugs (TAX), docetaxel, and anti‐HER‐2 agents such as trastuzumab. In an in vitro cell line study on drug‐sensitive MCF7, TAM‐resistant LCC‐2 cells, and TAX‐resistant MCF7 cells (MCF‐7/TAX), cells were treated with MET, TAX, and TAM against controls. Here, the results revealed MET‐induced activation of the HPP and inhibition of YAP in an AMPK‐independent route. The activation of classic upstream regulators of the HPP, KIBRA and FRDM6, were expressed in drug‐sensitive MCF7 cells upon treatment with MET. And further in drug resistant cells SCRIB mediated activation of HPP was observed. Interestingly, MET‐induced phosphorylation and cytoplasmic retention of YAP were observed in two different AMPK‐independent pathways by drug‐sensitive and drug‐resistant BC cells [37]. A schematic presentation of the anticancer mechanisms mediated by MET is presented in Figure 2 and the summary of literature evidence is tabulated in Table 1.

**TABLE 1:** Summary table of the anti‐cancer effects of Metformin on breast cancer cells.

Section	Mechanism	References
Adenosine monophosphate‐activated protein kinase (AMPK) mediated anticancer effects	Downregulates Metadherin (MTDH) which is an oncogenic protein	[26]
	Anti‐folate and tumor suppressor pathways mediated via 5‐formimino‐tetrahydrofolate and de novo purine and pyrimidine synthesis	[27]
	Downregulation of DVL3 and β‐catenin in the wnt/β‐catenin pathways, inhibiting cell cycle, ultimately resulting cell cycle arrest	[28]
	Negative regulation of mTOR in the tuberous sclerosis complex 1/2 (TSC1/2) mediated pathway	[29, 30]
	Combined treatment of MET with urosilic acid and MET with everolimus to results AMPK mediated inhibition of mTOR	[30, 31]
	AMPK with LKB‐1 has demonstrated the inhibition of epithelial mesenchymal transformation in a through TGF‐β and SMAD mediated pathway	[32, 33]
	Foxo3α and OSU‐53 mediated suppression of epithelial mesenchymal transformation	[24]
	Phenotypic polarization of macrophage population was shifted towards non‐malignant sub population	[34]
	Inhibits the express hypoxia‐inducible factor‐1α (HIF‐1α) which promotes vascularization in cancer tissues	[35]
	AMPK indirectly phosphorylate and induce cytoplasmic retention of yes‐associated protein inhibiting cell growth and proliferation in malignant tissues	[36]
Anti‐proliferation and inhibition of cell migration	Downregulate the expression of Wnt3a, Wnt5a, and β‐catenin have been significantly down regulated	[40]
	Reducing the expression of cell cycle biomarkers cyclin D1 (CD1) and proliferating cell nuclear antigen (PCNA)	[37]
	Downregulation of biomarkers of metastasis such as matrix metalloproteases (MMP)	[42]
	Impaired cell migration in vitro	[19]
Alteration of tumor micro‐environment	MET attenuate the malignant effect of Adipose derived stromal cells	[19]
	MET improves the effectiveness of ultrasound treatment for BC	[45]
Synergetic effects	Nano‐encapsulation of MET with doxorubicin to result synergistic anti‐cancer effects	[49, 50]
	Cisplatin and MET combination reduce cancer progression by downregulating RAD51	[57]
	MET exhibits synergistic effects with Cyclophosphamide (CP)	[60]
	MET together with tamoxifen and photosensitizers have collectively demonstrated improved anticancer effects	[61]
	The combination of MET and BMS‐754807 results cell cycle arrest by accumulating p27 via Skp2 mediated pathways	[62]
	MET, acetylsalicylic acid, oseltamivir phosphate and TAM synergistically induce anti‐cancer effects on triple negative BC cells and is evident with lower CD markers	[63, 64]
	Tangerine and MET synergistically induce accumulation of ROS in BC cells in vitro	[66]
	Sulforaphane and nano‐MET together reduce the cancer stem cell signaling and CD44 mediated carcinogenic effects	[68]
	MET in combination with TAM, Trastuzumab, and anti‐ROR1 downregulate proliferation, colony formation, migration, invasion, and tumor growth	[65]
	MET and rapamycin induce cell cycle arrest	[69]
	MET and 2‐D‐deoxy‐glucose, targeting glycolysis, synergized with chemotherapy	[70]
Apoptosis and ferroptosis	MET induce apoptosis markers namely HRK, TNFRSF10A and TNFRSF10B indicating apoptosis induction by MET	[40]
	MET induces ferroptosis and mediate ROS accumulation, inhibition of SLC7A11 expression in BC cells	[73]
Other	Tamoxifen and paclitaxel resistance has been overcome due to the combination of these drugs with MET	[37]

Despite its growing promise, the repurposing of metabolic drugs as anticancer agents remains constrained by several limitations. A major concern in many in vitro investigations of MET is the use of concentrations that far exceed those achievable under clinical conditions, often in the millimolar range, whereas plasma concentrations in MET‐treated patients typically remain within the micromolar range. This disparity largely reflects fundamental pharmacokinetic differences: in vitro systems lack the complex absorption, distribution, metabolism, and elimination processes that determine drug bioavailability and tissue distribution in vivo.

The rationale for employing supraphysiological doses stems from the hypothesis of mitochondrial accumulation of MET; however, definitive evidence for such accumulation in vivo remains lacking. Consequently, direct antiproliferative effects observed at high in vitro concentrations may not accurately translate to clinical settings. Moreover, MET's anticancer activity in vivo is likely multifactorial, involving systemic metabolic modulation that cannot be fully replicated in cell culture models.

Therefore, while in vitro studies provide valuable mechanistic insights, their findings must be interpreted with caution. Integration of pharmacokinetic data with robust in vivo and clinical evidence is essential to achieve a comprehensive understanding of MET's true therapeutic potential in oncology.

## 3 Conclusion

Metformin, long recognized as a cornerstone therapy for type 2 DM, is now emerging as a promising candidate in cancer therapeutics, particularly for BC. Evidence from extensive preclinical research indicates that MET exerts multifaceted anticancer effects through both AMPK‐dependent and AMPK‐independent mechanisms, including inhibition of cell proliferation, induction of apoptosis and ferroptosis, modulation of the tumor microenvironment, and suppression of metastatic pathways. Its ability to enhance the efficacy of conventional chemotherapeutic and endocrine agents further underscores its translational potential.

Taken together, current findings position MET not as an established anticancer therapy but as a compelling agent that warrants rigorous clinical investigation. Future well‐designed trials incorporating biomarker‐guided patient selection and pharmacokinetic profiling are essential to define its optimal role in breast cancer prevention and treatment.

## Author Contributions

**Ambulugala Gamage Rajika Greshamali Jinadasa:** methodology, writing – original draft. **N. D. Amal Wageesha:** writing – review and editing, supervision. **Sameera R. Samarakoon:** supervision. **Sagarika Ekanayake:** writing – review and editing, supervision. **H. M. Kasuni Akalanka:** conceptualization, supervision, writing – review and editing, funding acquisition.

## Conflicts of Interest

The authors declare no conflicts of interest.

## Data Availability

The data that support the findings of this study are available from the corresponding author upon reasonable request.
